# Mathematical modeling reveals the factors involved in the phenomena of cancer stem cells stabilization

**DOI:** 10.1371/journal.pone.0224787

**Published:** 2019-11-11

**Authors:** Nikolay Bessonov, Guillaume Pinna, Andrey Minarsky, Annick Harel-Bellan, Nadya Morozova

**Affiliations:** 1 Institute of Problems of Mechanical Engineering, Russian Academy of Sciences, Saint-Petersburg, Russia; 2 Institute for Integrative Biology of the Cell (I2BC), CEA, CNRS, University Paris‐Sud, University Paris‐Saclay, Gif‐sur‐Yvette, France; 3 Saint-Petersburg Academic University, Russian Academy of Sciences, Saint-Petersburg, Russia; 4 Institut des Hautes Etudes Scientiques (IHES), Bures-sur-Yvette, France; University of Maryland, UNITED STATES

## Abstract

Cancer Stem Cells (CSC), a subset of cancer cells resembling normal stem cells with self-renewal and asymmetric division capabilities, are present at various but low proportions in many tumors and are thought to be responsible for tumor relapses following conventional cancer therapies. In vitro, most intriguingly, isolated CSCs rapidly regenerate the original population of stem and non-stem cells (non-CSCs) as shown by various investigators. This phenomenon still remains to be explained. We propose a mathematical model of cancer cell population dynamics, based on the main parameters of cell population growth, including the proliferation rates, the rates of cell death and the frequency of symmetric and asymmetric cell divisions both in CSCs and non-CSCs sub-populations, and taking into account the stabilization phenomenon. The analysis of the model allows determination of time-varying corridors of probabilities for different cell fates, given the particular dynamics of cancer cells populations; and determination of a cell-cell communication factors influencing these time-varying probabilities of cell behavior (division, transition) scenarios. Though the results of the model have to be experimentally confirmed, we can anticipate the development of several fundamental and practical applications based on the theoretical results of the model.

## Introduction

Stem cells are undifferentiated cells present in very low numbers in most tissues. Stem cells are responsible for tissue renewal and homeostasis, by giving rise to non-stem cells that proliferate and further differentiate in specialized cells. Stem cells show very specific features, notably regarding cell division: they are able to undergo asymmetrical division, dividing into a stem cell and non-stem cell; moreover, the rate of stem cells division is very low as compared to that of non-stem cells [1–3].

It has been demonstrated that in most malignant tumors, cancer cell populations appear to include a rare stem cell-like subpopulation suspected to be responsible for the initiation and maintenance of tumors in animals [4–14]. This subpopulation can be detected and purified using specific cellular probes or cell surface markers. *In vitro*, purified cancer stem cells (CSCs) are able to reconstitute the population heterogeneity whereas, in contrast, purified non-stem cells cannot. Also, CSCs were shown to be highly tumorigenic in xenografts experiments, and to be responsible for cancer metastasis, i. e. colonization of various tissues by the primary tumor. Because of these features, cancer stem cells are also called tumor-initiating cells [7]. However, not all cancer stem cells appear to have all the features of normal stem cells. For example, CSCs may have a diminished capacity to undergo asymmetrical division compared to normal stem cells [6,12,15–17].

CSCs have been demonstrated in most solid and hematologic tumors [8,10,16,18–28], with very well described common functional features, i. e. an indefinite self-renewal capability and the ability to undergo some asymmetric divisions. Importantly, CSCs are resistant to chemo- and radio- therapies [4,5,9,10,29–31], suggesting that they may be responsible for tumor relapses following chemo- or radio-therapy. This has important implications in therapeutic, as most of the current treatments target a regression of the tumors mass without accounting for the tumor functional heterogeneity.

Various mathematical models have been proposed for describing the dynamics of both normal [32–36] and cancer [30,31,37–47] stem cell populations behavior. These works suggest two different concepts for description of stem cells population behavior. One concept is based on the principle that stem cells act according to their intrinsic program, which may be deterministic or stochastic [30,31,35,39–41,45,48]. Alternatively, a concept of self-organization of stem cells suggests modeling of the entire system of cell-cell and cell-environment interactions, for which some authors also consider a stochastic behavior [35,36,49–51]. Modeling of cancer cell population behavior provides very important inferences as for understanding the nature of cancer growth so for clinical prognosis and treatment strategies. In many cases it allows to have evidence about factors that cannot be measured directly in clinical or in experimental investigations. For example, due to the mathematical model it was shown that the role of leukemia stem-like cells population on the course of disease is much greater than the one of leukemia blast cells [42,43,47]. The model-based estimation of prognostic factors in clinical data may help in designing treatment strategies [42–47]. Moreover, the quantitative information about the cancer stem cells (CSC) or tumor-initiating cells (TIC) fraction dynamics can be inferred by methods based on mathematical models. The method described in [44] identifies two characteristic equilibrium TIC regimes during expansion and regression of chronic myeloid leukemia.

However, the possibility of dedifferentiation of non-stem cancer cell to stem cell is one of the most important and yet unsolved question about CSC behavior, which has been, so far, addressed in only few of these works [30,38,52]. These (stochastic) models base their principle on the assumption that all possible transitions in subpopulations can occur spontaneously, with some probabilities.

**Fig 1:**
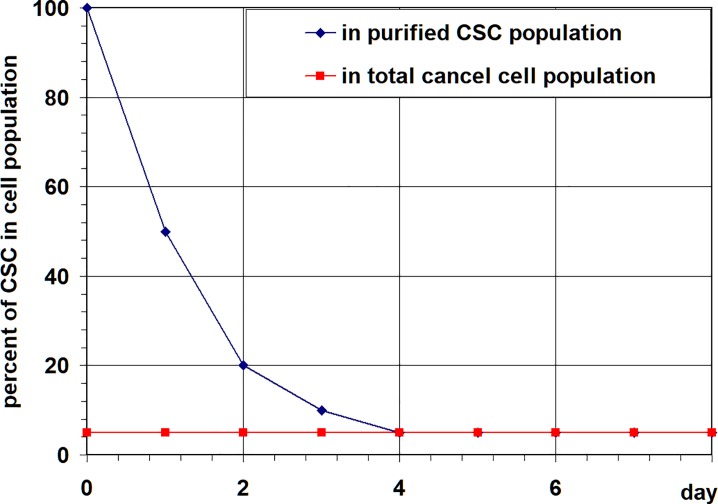
Stabilization of Cancer Stem Cells population in cell culture. Schematic curves showing a percentage of CSC over time (summarized from numerous published and unpublished data). Dotted red curve: a basal level of CSC percentage, constant over years of cell passages; dark blue curve: dynamics of isolated cancer stem cell population up to stabilization at characteristic level of CSC percentage.

Also, only few modeling approaches were proposed to gain insight into the intriguing *phenomena of cancer stem cells population stability* [38,44,53,54]. This *phenomena* detected in many cancer cell lines harboring measurable levels of cells with CSC features, is that over several years of cell passage the relative number of cancer stem cells fluctuates around a basal level, characteristic for each specific cell line (as illustrated in Fig 1, dotted red curve). Moreover, it has been shown that isolated cancer stem cells can rapidly regenerate in culture the heterogeneity of the parental cell line with the characteristic relative percentage of cancer stem cells (as illustrated in Fig 1, dark blue curve).

One work discussing this phenomenon models the CSC behavior as a Markov process [38]. The model is based on stochasticity of single-cell behaviors and does not consider cell-to-cell communications.

In our previous work [53,54] we constructed and analyzed a mathematical model that takes into account this intriguing characteristic of CSC population behavior. We suggested an instructive role of cell-to-cell signaling influencing the cell parameters and leading to CSC population equilibrium. The mathematical model accounts for all possible cancer stem and non-stem cell behaviors, i. e. type of division (symmetric or asymmetric), direct transition (differentiation or dedifferentiation) and cell death. The analysis of the model helped to elucidate some important characteristics of cancer stem cells evolution, in particular, a set of parameters of cell growth implying the necessity of non-stem to stem cell transition.

In this work we expand this mathematical model and address the question of “instructive signal(s)” underlying the phenomena of cancer cell population stability, aiming to provide meaningful predictions on its dynamics and nature. In the presented work we continue analysis of the model aiming to solve the following problems:

- determination of time-varying corridors of probabilities of different cell fates, given the dynamics of cancer cells populations;

- determination of a cell-to-cell communication factors, influencing time-varying probabilities of cell behavior (division, direct transition) scenarios.

We demonstrate that using data measured in the context of CSC population stabilization, our model is able to infer corridors of time-varying probabilities of cancer cell fates that provide significant insights into the cellular dynamics of heterogeneous tumors. Next we show how the set of curves of probabilities can help identifying a set and kinetics of secreted factors responsible for cell population behavior.

## Methods

1. Algorithm for the solution of the system of Eqs (14–17).

The system can be rewritten in the form:

$$\frac{dp_{1}}{dt}=\frac{2\lambda_{1}(s-1)(\left(\lambda_{2}q_{2}(2+(s-2)s)-2\dot{s})\dot{s}+s(s((\gamma_{2}-\gamma_{1}+\lambda_{1}-\lambda_{2}+(p_{1}+2p_{2})\lambda_{1})\dot{s}-\ddot{s})+2({\dot{s}}^{2}+\ddot{s})\right))}{\left(s-2)(4\lambda_{2}^{2}{\left(1-s\right)}^{2}+2\lambda_{1}^{2}s^{2}(11+3s(s-2))\right)},$$ (A1)

$$\frac{dp_{2}}{dt}=\frac{2\lambda_{1}(s+3)(\left(\lambda_{2}q_{2}(2+(s-2)s)-2\dot{s})\dot{s}+s(s((\gamma_{2}-\gamma_{1}+\lambda_{1}-\lambda_{2}+(p_{1}+2p_{2})\lambda_{1})\dot{s}-\ddot{s})+2({\dot{s}}^{2}+\ddot{s})\right))}{\left(s-2)(4\lambda_{2}^{2}{\left(1-s\right)}^{2}+2\lambda_{1}^{2}s^{2}(11+3s(s-2))\right)},$$ (A2)

$$p_{3}=\frac{\dot{s}-\lambda_{2}q_{2}+(\gamma_{1}-\gamma_{2}+\lambda_{1}+\lambda_{2}–\lambda_{1}p_{1}+\lambda_{2}q_{2}+(\gamma_{2}-\gamma_{1}-\lambda_{2}+\lambda_{1}(p_{1}+p_{2}))s)s}{\lambda_{1}s(2–s)}$$ (A3)

$$p_{4}=1-p_{1}-p_{2}-p_{3},$$ (A4)

$$q_{1}=1-q_{2},$$ (A5)

$$\frac{dq_{2}}{dt}=\frac{4\lambda_{2}(s-1)(\left(\lambda_{2}q_{2}(2+(s-2)s)-2\dot{s})\dot{s}+s(s((\gamma_{2}-\gamma_{1}+\lambda_{1}-\lambda_{2}+(p_{1}+2p_{2})\lambda_{1})\dot{s}-\ddot{s})+2({\dot{s}}^{2}+\ddot{s})\right))}{s(s-2)(4\lambda_{2}^{2}{\left(1-s\right)}^{2}+2\lambda_{1}^{2}s^{2}(11+3s(s-2)))},$$ (A6)

where s ≡ s(t), $\dot{s}\equiv \frac{ds(t)}{dt}$, and $\ddot{s}\equiv \frac{d^{2}s(t)}{dt^{2}}$, are known functions, p_i_,q_j_ (i = 1…4,j = 1,2), are unknown variables.

A system (A1-A6) was approximated by a second order finite difference scheme

$$\frac{p_{1}^{n+1}-p_{1}^{n}}{\Delta t}=\frac{2\lambda_{1}(s-1)(\left(\lambda_{2}q_{2}^{n+1/2}(2+(s-2)s)-2\dot{s})\dot{s}+s(s((\gamma_{2}-\gamma_{1}+\lambda_{1}-\lambda_{2}+(p_{1}^{n+1/2}+2p_{2}^{n+1/2})\lambda_{1})\dot{s}-\ddot{s})+2({\dot{s}}^{2}+\ddot{s})\right))}{\left(s-2)(4\lambda_{2}^{2}{\left(1-s\right)}^{2}+2\lambda_{1}^{2}s^{2}(11+3s(s-2))\right)},$$ (A7)

$$\frac{p_{2}^{n+1}-p_{2}^{n}}{\Delta t}=\frac{2\lambda_{1}(s+3)(\left(\lambda_{2}q_{2}^{n+1/2}(2+(s-2)s)-2\dot{s})\dot{s}+s(s((\gamma_{2}-\gamma_{1}+\lambda_{1}-\lambda_{2}+(p_{1}^{n+1/2}+2p_{2}^{n+1/2})\lambda_{1})\dot{s}-\ddot{s})+2({\dot{s}}^{2}+\ddot{s})\right))}{\left(s-2)(4\lambda_{2}^{2}{\left(1-s\right)}^{2}+2\lambda_{1}^{2}s^{2}(11+3s(s-2))\right)},$$ (A8)

$$p_{3}^{n+1}=\frac{\dot{s}-\lambda_{2}q_{2}^{n+1}+(\gamma_{1}-\gamma_{2}+\lambda_{1}+\lambda_{2}–\lambda_{1}p_{1}^{n+1}+\lambda_{2}q_{2}^{n+1}+(\gamma_{2}-\gamma_{1}-\lambda_{2}+\lambda_{1}(p_{1}^{n+1}+p_{2}^{n+1}))s)s}{\lambda_{1}s(2–s)},$$ (A9)

$$p_{4}^{n+1}=1-p_{1}^{n+1}-p_{2}^{n+1}-p_{3}^{n+1},$$ (A10)

$$q_{1}^{n+1}=1-q_{2}^{n+1},$$ (A11)

$$\frac{q_{2}^{n+1}-q_{2}^{n}}{\Delta t}=\frac{4\lambda_{2}(s-1)(\left(\lambda_{2}q_{2}^{n+1/2}(2+(s-2)s)-2\dot{s})\dot{s}+s(s((\gamma_{2}-\gamma_{1}+\lambda_{1}-\lambda_{2}+(p_{1}^{n+1/2}+2p_{2}^{n+1/2})\lambda_{1})\dot{s}-\ddot{s})+2({\dot{s}}^{2}+\ddot{s})\right))}{s(s-2)(4\lambda_{2}^{2}{\left(1-s\right)}^{2}+2\lambda_{1}^{2}s^{2}(11+3s(s-2)))},$$ (A12)

where $p_{i}^{n+1/2}=\frac{p_{i}^{n+1}+p_{i}^{n}}{2},(i=1\ldots 4)$, $q_{j}^{n+1/2}=\frac{q_{j}^{n+1}+q_{j}^{n}}{2},(j=1,2)$, Δt is a time step, n is a time step number.

We consider that the initial conditions:

$$p_{1}(t=0)=p_{10},\;p_{2}(t=0){=p}_{20},\;q_{2}(t=0)=q_{20}$$ (A13)

are given.

Then, the initial values for $p_{i}^{0},\;q_{j}^{0},\;(i=1\ldots 4,j=1,2)$ are set by (A13) and the calculations from (A3-A5), using (A13). By that, from numerical solution of the system (A7-A12) we can obtain $p_{i}^{n+1},\;q_{j}^{n+1}\;(i=1\ldots 4,j=1,2)$ for any time step n = 1,2,…. Accuracy of numerical simulations is controlled by decreasing time step.

Thus a triple of parameters p_10_, p_20_, q_20_ allows obtaining $p_{i}^{n},\;q_{j}^{n}$ for any time step. If at some time step the conditions

$$0\leq p_{i}^{n}\leq 1,\;i=1\ldots 4;\;0\leq q_{j}^{n}\leq 1,\;j=1,2,$$ (A14)

were violated, the corresponding triple of parameters p_10_, p_20_, q_20_ was discarded and a new one was selected from the interval [0,1]. The consequent repeating of this simulation for all possible combinations of parameters p_10_, p_20_, q_20_, starting from the triple (0,0,0) up to the triple (1,1,1) with a step 0,01 for each parameter p_10_, p_20_, q_20_ allowed detecting the intervals of initial conditions:

$$p_{10}^{min}\leq p_{10}\leq p_{10}^{max},\;p_{20}^{min}\leq p_{20}\leq p_{20}^{max},\;q_{20}^{min}\leq q_{20}\leq q_{20}^{max},$$

which satisfy (A14) for all time period T and thus provide a desired corridors of all probabilities.

A user interface is developed allowing to input the parameters of the model and to output the results in the graphical form and in the form of numerical files.

The corresponding program code can be found at:

https://github.com/nickbessonov/CSC-article/blob/master/CSC%20(17.05.18).rar)

2 Algorithm for the solution of the system of Eq (25).

The system of Eq (25) was solved numerically by well known trick when the system of steady equations was replaced by a system of simplest non-steady equations and the solution of a steady problem is sought by solving the non-steady problem and finding its steady solution.

In our case, for determining b_ik_,a_k_,c_k_, k = 1…K, i = 1…6, the system Eq (25) was replaced by a system of the next non-steady equations

$$\left\{ \begin{array}{c} \frac{a_{q}^{j+1}-a_{q}^{j}}{\Delta t_{q}}=-2\sum_{n=1}^{N}\left(e^{-a_{q}^{j}{\left(t_{n}-c_{q}^{j}\right)}^{m}}{\left(t_{n}-c_{q}^{j}\right)}^{m}\sum_{i=1}^{6}b_{iq}^{j}(\sum_{k}^{K}\left(b_{ik}^{j}e^{-a_{k}^{j}{\left(t_{n}-c_{k}^{j}\right)}^{m}}\right)-y_{in})\right)\\ \frac{c_{q}^{j+1}-c_{q}^{j}}{\Delta t_{c}}=2a_{q}m\sum_{n=1}^{N}(e^{-a_{q}^{j}{\left(t_{n}-c_{q}^{j}\right)}^{m}}{\left(t_{n}-c_{q}^{j}\right)}^{\left(m-1\right)}\sum_{i=1}^{6}b_{iq}^{j}(\sum_{k=1}^{K}\left(b_{ik}^{j}e^{-a_{k}^{j}{\left(t_{n}-c_{k}^{j}\right)}^{m}}\right)-y_{in})),\\ \frac{b_{iq}^{j+1}-b_{iq}^{j}}{\Delta t_{b}}=2\sum_{n=1}^{N}\left(e^{-a_{q}^{j}{\left(t_{n}-c_{q}^{j}\right)}^{m}}(\sum_{k=1}^{K}\left(b_{ik}^{j}e^{-a_{k}^{j}{\left(t_{n}-c_{k}^{j}\right)}^{m}}\right)-y_{in})\right) \end{array}\right.$$ (B1)

where Δt_q_, Δt_c_, Δt_b_ are pseudo time step, j is number of pseudo time step.

The system (B1) was solved with the help of iterations. Trial initial values $a_{q}^{0},\;c_{q}^{0}$, and $b_{iq}^{0}$ q = 1…K, i = 1…6 were set. Substituting these values into the right-hand side of the Eq (B1), we determined the values of unknown parameters in the next pseudo time step $a_{q}^{1},\;c_{q}^{1}$, and $b_{iq}^{1}$. Then this process was repeated until the required accuracy of the solution was reached. The only one difficulty arises at the beginning of application of this trick when it is necessary to determine the signs of time steps. This problem was solved easy by several numerical experiments. The sign minus in right part of (B1) is a result of these computational experiments. The corresponding program code can be found at:

https://github.com/nickbessonov/CSC-article/blob/master/CSC%20(17.05.18).rar)

3 Experimental part

The measurements of cell division rates were performed on several human cancer cell lines, namely five NSCLC (non-small cell lung cancer) cell lines (A549,HCC827, H1299, H1650, H1975), four ovarian cancer cell lines (A2780, HEY, OV2008, TOV-112D cells) and colon carcinoma HCT 116 cell line. The detected proliferation rates of non-stem cells were similar or considerably close in all investigated cell lines, thus its averaged value was taken for the numerical simulations.

The percentage of cell death rates was measured by Fluorescence Activated Cell Sorting (FACS) analysis in A549 cell line at every day during 10 days after passage. The dead cells were stained by 7 AAD (7-AminoActinomycin D) and sorted accordingly. The analysis have shown the rate of cell death around 0.1 (10% of all cells) for all days during the period of cultivation. The second variant of death rate (0.5) was added to the numerical simulations to explore another possibility of death rate, close to a critical one, which was noticed in another investigated cell lines (see above), but without precise measurements.

## Results and discussion

### Mathematical model

Following our previous work [53] we suggest a model accounting for 4 scenarios of cell behaviors (different types of cell divisions and direct transition) for stem and non-stem cells, and assumed that each scenario can occur with some probability (Table 1):

**Table 1:** Possible scenarios of cell behavior. S = Stem cell; D = Daughter cell.

Stem cell(S)		Daughter cell (D)	
scenario	probability	scenario	probability
*S*→*S*+*D*	*p*_1_	*D*→*D*+*D*	*q*_1_
*S*→*D*+*D*	*p*_2_	*D*→*S*+*D*	*q*_2_
*S*→*S*+*S*	*p*_3_	*D*→*S*+*S*	*q*_3_
*S*→*D*	*p*_4_	*D*→*S*	*q*_4_

Though we include the scenarios *D*→*S*+*S* and *D*→*S* for daughter cells in Table 1 as theoretically possible, for a time being we will consider in a model only two scenarios for daughter cells, which discriminates two principal modes of their behavior: the regular one (*D*→*D*+*D*) and the de-differentiation of daughter cells into S cells, which requires the activation of different genetic program. For the representation of the last case we consider only one scenario *D*→*S*+*D* (for which we assume the transition *D*→*S* as a part of it, meaning that D cell should undergo a transition *D*→*S* before the next asymmetric division). Thus, for the current model the probabilities q_3_ and q_4_ are taken to be equal to 0.

The probabilities should satisfy the usual restrictions on probabilities:

$$0\leq p_{i}\leq 1,\;i=1\ldots 4;\;0{\leq q}_{i}\leq 1,\;i=1,2,$$ (1)

$$p_{1}+p_{2}+p_{3}+p_{4}=1,$$ (2)

$$q_{1}+q_{2}=1.$$ (3)

We considered that cell divisions/transition occur with the rate λ_1_ in stem cells (S) and with the rate λ_2_ in daughter cells (D), and that death rates are γ_1_ and γ_2_ in S and D cells respectively. We need to comment that though it seems more plausible to consider that each mode of cell divisions or transition occurs with its own rate (e.g., like in models [42,43], we will consider in our model one constant rate of cell divisions (or direct transition) for stem cells, and another constant rate of cell divisions for non-stem cells, independently from a scenario. Also, though it was shown that in general cell division rates may change in time, for example, due to regulation by signaling factors [55–57], we assume the possibility to consider them to be constant during the short period of stabilization of cell population. The same assumption is made in many mathematical models addressing the cancer cells population behavior [42–47].

The proposition (Table 1) gives a system of differential equations for the dynamics of *S* and *D* cancer cells:

$$\left\{ \begin{array}{c} dS(t)/dt=\alpha_{S}S(t)+\beta_{D}D(t)\\ dD(t)/dt=\beta_{S}S(t){+\alpha }_{D}D(t) \end{array}\right.$$ (4)

with coefficients, depending on probabilities of scenarios p_i_ and q_i_, and on parameters λ_1_, λ_2_, γ_1_, γ_2_ (growth and death rates):

$$\begin{array}{c} \alpha_{S}=((p_{1}+2p_{3}-1)\lambda_{1}-\gamma_{1}),\;{\beta_{D}=q}_{2}\lambda_{2},\\ \beta_{S}=(p_{1}+{2p}_{2}+p_{4})\lambda_{1}S(t),\;\alpha_{D}=(q_{1}\lambda_{2}-\gamma_{2}). \end{array}$$ (5)

Using (5), the system (4) can be re-written as:

$$\left\{ \begin{array}{c} dS(t)/dt=((p_{1}+2p_{3}-1)\lambda_{1}-\gamma_{1})S(t)+q_{2}\lambda_{2}D(t)\\ dD(t)/dt=(p_{1}+{2p}_{2}+p_{4})\lambda_{1}S(t)+(q_{1}\lambda_{2}-\gamma_{2})D(t) \end{array}\right..$$ (6)

In our previous work, using the system (6), we have analyzed the time-dependent evolution and the asymptotic behavior of the percentage of cancer stem cells *s*(*t*) in a cancer cell population with the initial conditions:

$$S(t=0)=S_{0},\;D(t=0)=0$$ (7)

corresponding to the dynamics of isolated cancer stem cells population up to stabilization, as illustrated in Fig 1.

The fraction (percentage) of stem cells is calculated as:

s(t)=S(t)/(S(t)+D(t)). (8)

### Corridors of probabilities of cell fate scenarios

Here we continue analysis of the model aiming to determine the time-varying corridors of probabilities of different cell fates, given the dynamics of cancer cells populations. We assume that a curve s(t) of the percentage of CSC over time displaying a CSC stabilization after perturbation (as shown at Fig 1), and proliferation and death rates of cells are given as a result of experimental measurements in a particular cell line.

First, our numerical simulations show that a good fitting of model (6) to any reference curve s(t) (determined by the least squares method, with an average deviation equal ~ 5% or less) can be achieved in admission that the set of parameters λ_i_,γ_i_,p_i_,q_i_ changes over time. As discussed in Section 1, we may admit for the following analysis that the parameters changing with time should be the probabilities of scenarios *p*_*i*_(*t*),*q*_*i*_(*t*), while parameters λ_i_, γ_i_, may be considered as constant.

This led us to *Question 1*:

*Given the dynamics of percentage of CSC s(t)*, *is it possible to find functions* p_i_(*t*), i = 1,2,3,4, q_i_(*t*), *i* = 1,2 *for a given constant set of* λ_i_ *and* γ_i_?

In order to solve this problem we first considered the following hypothesis: *all changes in cell behavior scenarios up to stabilization should be minimized (the sum of all changes of p*_*i*_(*t*) *and q*_*i*_(*t*) *should be the minimal possible ones)*. The underlying biological logic of this hypothesis is that each type of scenario is implemented by different sets of biological mechanisms, which require specific proteins and other compounds to be prepared in a cell. This means that each time-step of cell population behavior towards stabilization should be more plausibly achieved by minimal change in each scenario existing at the previous time-step than by intermittent pattern of each of them.

This means that the function v:

$$v={\left({\dot{p}}_{1}\right)}^{2}+{\left({\dot{p}}_{2}\right)}^{2}+{\left({\dot{p}}_{3}\right)}^{2}+{\left({\dot{p}}_{4}\right)}^{2}+{\left({\dot{q}}_{1}\right)}^{2}+{\left({\dot{q}}_{2}\right)}^{2},$$ (9)

where ${{\dot{p}}_{i}=dp}_{i}(t)/dt,\;{{\dot{q}}_{i}=dq}_{i}(t)/dt$, should be minimized, i.e.:

$$v={\left({\dot{p}}_{1}\right)}^{2}+{\left({\dot{p}}_{2}\right)}^{2}+{\left({\dot{p}}_{3}\right)}^{2}+{\left({\dot{p}}_{4}\right)}^{2}+{\left({\dot{q}}_{1}\right)}^{2}+{\left({\dot{q}}_{2}\right)}^{2}\to min.$$ (10)

Next we rewrite the system (6) and the conditions (2) and (3) as:

$$\begin{array}{c} \dot{s}(t)=q_{2}(t)\lambda_{2}+\left((p_{3}(t)-p_{2}(t)-p_{4}(t))\lambda_{1}-(1+q_{2}(t))\lambda_{2}+\gamma_{2}-\gamma_{1}\right)s(t)\\ +(\lambda_{2}-(1-p_{4}(t))\lambda_{1}-\gamma_{2}+\gamma_{1})s^{2}(t), \end{array}$$ (11)

$$p_{1}(t)+p_{2}(t)+p_{3}(t)+p_{4}(t)=1,$$ (12)

$$q_{1}(t)+q_{2}(t)=1,$$ (13)

where $\dot{s}(t)=ds(t)/dt$.

This gives us a system:

$$\begin{array}{c} p_{3}(t)=\{\dot{s}(t)-\lambda_{2}q_{2}(t)+[\gamma_{1}-\gamma_{2}+\lambda_{1}+\lambda_{2}–\lambda_{1}p_{1}(t)+\lambda_{2}q_{2}(t)\\ +(\gamma_{2}-\gamma_{1}-\lambda_{2}+\lambda_{1}(p_{1}(t)+p_{2}(t)))s(t)]s(t)\}/[\lambda_{1}s(t)(2–s(t))], \end{array}$$

$$p_{4}(t)=1-p_{1}(t)-p_{2}(t)-p_{3}(t),$$ (14)

$$q_{1}(t)=1-q_{2}(t)$$

from which we can conclude that the function v depends only upon three variables, which are (for example) p_1_(*t*),p_2_(*t*),q_2_(*t*). Thus, for minimizing it, we need to solve 3 equations:

$$\frac{\partial v}{\partial {\dot{p}}_{1}}=0,$$ (15)

$$\frac{\partial v}{\partial {\dot{p}}_{2}}=0,$$ (16)

$$\frac{\partial v}{\partial {\dot{q}}_{2}}=0.$$ (17)

Thus, for six variables p_i_(*t*) and q_i_(*t*) we have a system of 6 Eqs (14–17). For the solution of that system we have to add three initial conditions:

$$p_{1}(t=0)=p_{10},\;p_{2}(t=0){=p}_{20},\;q_{2}(t=0)=q_{20}.$$ (18)

All solutions for each p_i_(*t*), q_i_(*t*) corresponding to all possible sets of initial conditions (18), which satisfy the condition (1) for all time period T will provide a set of six corridors (ranges) of probabilities of scenarios p_i_(*t*), q_i_(*t*) of cell behavior in a given experimental case.

This means that, *given the measured s(t) and a constant set of* λ_i_,γ_i_, *we can determine corridors of all possible probabilities of scenarios* p_i_(*t*), q_i_(*t*) *varying with time*.

**Fig 2:**
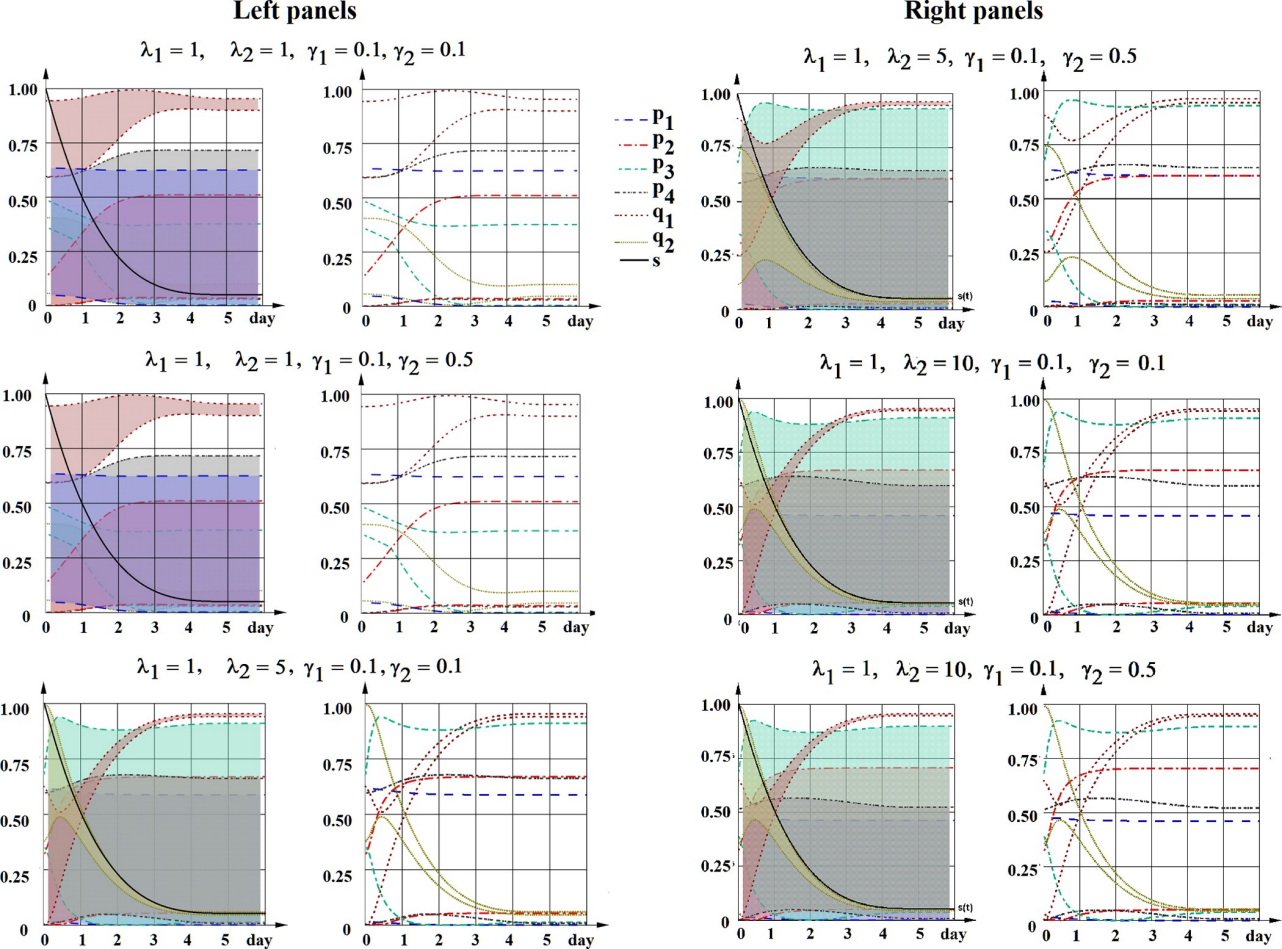
The ranges (corridors) of all possible solutions for probabilities p_i_, q_i_ for the reference curve of CSC sub–population dynamics (s(*t*), black curve). The boundaries of corridors are determined by satisfying the condition (1). Left panels: each “corridor of probability” is shown in sheeted color. The reference curve of CSC sub–population dynamics s(*t*) is shown in light green color. Right panels: “corridor of probability” is marked by max and min colored lines for the following sets of λ_1_,λ_2_,γ_1_,γ_2_.

The results of the calculations of all possible corridors of probabilities for the reference curve s(t) (taken as the one in Fig 1), and different sets of biologically relevant parameters λ_1_,λ_2_,γ_1_,γ_2_ are presented in Fig 2; the corresponding algorithm and the program code are provided in Methods section. The considered rates of non-stem cell division (λ_2_) were taken from experimental measurements in different cancer cell lines, while the rate of stem cell division (λ_1_), which is difficult to measure, was chosen in order to vary the ratio of stem/non-stem division rates (1:1; 1:5; 1:10), corresponding to a statement about slower rate of stem cells divisions. The considered variants of cell death rates (0.1; 0.5) were taken from experimental measurements.

It is important to note, that the boundaries of each corridor can be (and usually are) comprised from the parts of different possible functions p_i_(*t*) (or q_i_(*t*)) existing inside a particular corridor. However, these boundaries can help to evaluate the possibility or impossibility of a scenario(s) in particular experimental systems.

For example, this simulation gives an answer to the question of the *necessity of non-stem to stem cell transition scenario* in the course of CSC stabilization toward equilibrium. As it can be seen from the Fig 2, for all considered biologically relevant sets of cell division and cell death rates λ_1_,λ_2_,γ_1_,γ_2_, the lowest boundary of the corridor of probability q_2_(*t*) (corresponding to non-stem to stem cell transition) appears to be higher than zero, at all or at least at some time points.

Another important question is the existence of other types of stem cell behavior scenarios in addition to asymmetrical division, which is postulated to be predominant at least in normal stem cells. Results showed that for biologically relevant sets of parameters λ_1_,λ_2_,γ_1_,γ_2_, the highest level of possible probability p_i_(*t*) (the upper corridor boundary) is around 60%, being much lower for some cases (Fig 2).

For many practical applications it is very important to be able to find a unique solution for each probability p_i_(*t*), q_i_(*t*). To this end, we addressed *Question 2*:

*Given a measured curve s*(*t*), *and parameters* λ_i_,γ_i_, *what additional data are necessary and sufficient to get a unique solution for each* p_i_(*t*) and q_i_(*t*)?

From the analysis done for the Question 1 it is clear that these additional data should be a set of three initial conditions (18).

**Fig 3:**
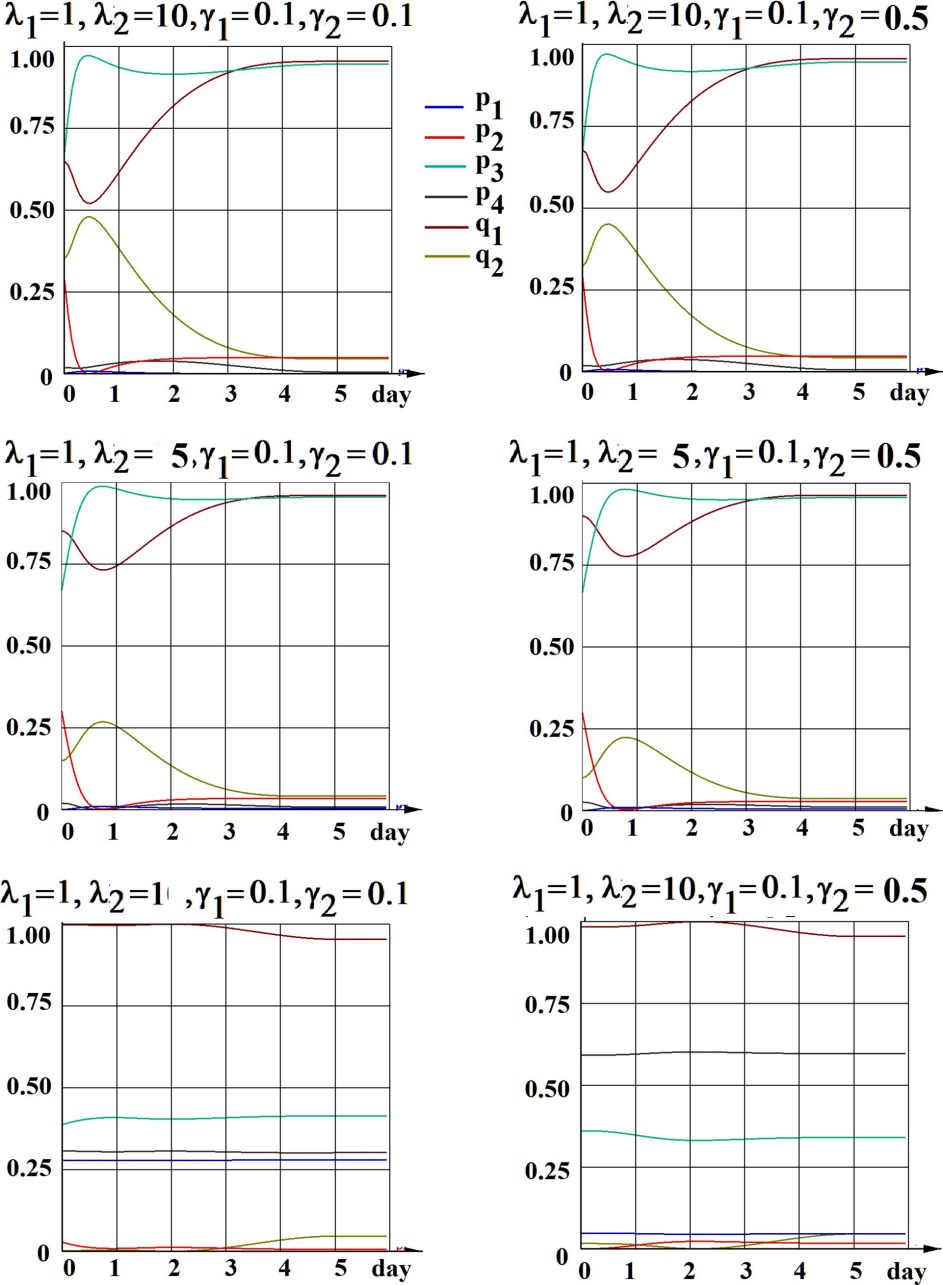
The unique solutions for each function p_i_(*t*) and q_i_(*t*) determined by the choice of initial conditions (18) requesting an absence (maximal possible minimization) of *D*→*S* scenario (*q*_20_→min). Six different sets of parameters λ_i_,γ_i_, (as in Fig 2) are considered.

**Fig 4:**
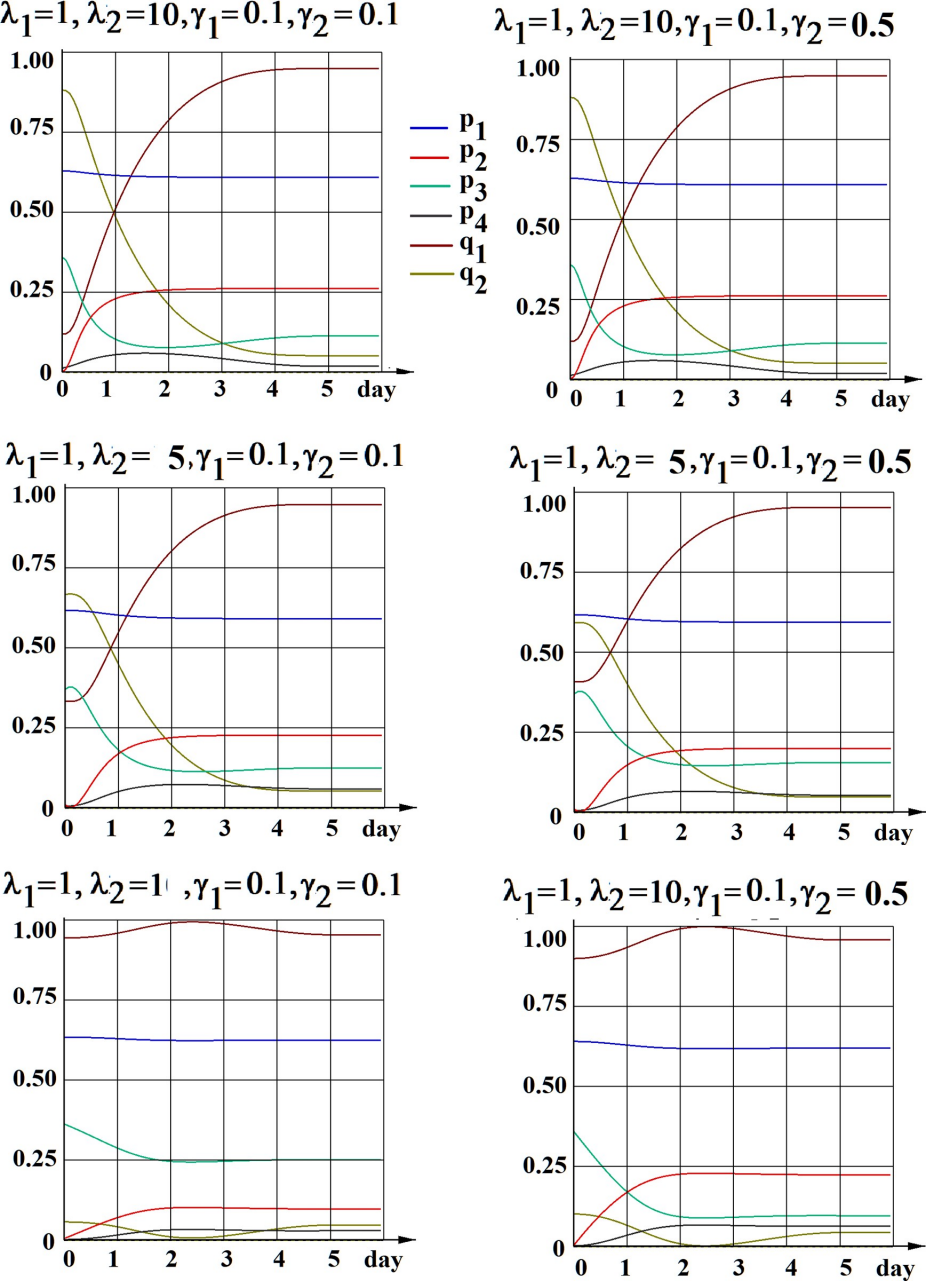
The unique solutions for each function p_i_(*t*) and q_i_(*t*) determined by the choice of initial conditions (18) requesting the maximal probability of asymmetric division of stem cells (*p*_10_→max). Six different sets of parameters λ_i_,γ_i_, (as in Fig 2) are considered.

We have explored this possibility and present two examples of the determination of such a set of unique functions p_i_(*t*) and q_i_(*t*) in Figs 3 and 4, considering two important biological questions, mentioned above. In the first simulation (Fig 3) we chose the initial conditions (18) such that *q*_20_ be the minimal one, thus exploring the possibility of an absence of D to S cell transition the scenario for each particular case (six different cases (sets of parameters λ_i_,γ_i_,as in Fig 2) are considered). As we can see from our results, this possibility does not occur in any of these cases. In the second simulation (Fig 4) we search for the maximal probability of asymmetric division of stem cells in each particular case, by setting the initial conditions to maximize *p*_10_. The results show that for all six cases the probability *p*_1_ can not exceed 63%.

### Determination of underlying field factors involved in cancer cell population dynamics

In our previous work [53] we have suggested that the coordinated dynamical change of the parameters of cell behavior, resulting in cancer stem cells subpopulation stabilization, occurs in response to a multiparametric biochemical signal produced in a system. In the simplest case, it may be a set of secreted factors influencing cell behavior; although it can also be induced by cell-to-cell contact, a hypothesis that is not taken into account here for the sake of simplification.

We noted this signal as an *underlying field* u(*t*), where u(*t*) is a set of biochemical compounds (factors) in a media, capable to influence cell fate. Generally, in can be presented as a matrix of factors u_*ij*_(*t*), where each factor *i* may be produced by S or D cell at some time point t, and have an influence *j* on the behavior of S or/and D cell, possibly with some time delay t+r.

Here we further explore this idea, assuming that a field of secreted factors u(*t*) influences probabilities of cell fate scenarios, and thus that the structure of corridors of probabilities p_i_ and q_i_ depends on a changing set of concentrations of secreted biochemical compounds.

This means that in the Eq (10) all probabilities of division scenarios of S and D cells (p_i_(*t*) and q_i_(*t*) are the functions of an underling field u(*t*), changing with time:

$$p_{i}(u(t)),\;i=1\ldots 4,\;q_{i}(u(t)),\;i=1,2.$$ (19)

The task of identification of molecular factors involved in the underlying field formation raises the *Question 3*:

*Given a set of unique functions of probabilities p*_*i*_ *and q*_*i*_, *is it possible to find a set of factors* u(*t*) *responsible for their dynamics?*

First, for simplification, we will consider *underlying field* u(*t*) merely be a set of K biochemical compounds (factors) presenting in the media, i.e., as a set of functions *u*_*k*_(*t*), k = 1,…,K. Later, we may consider the possible dependence of each factor u_*k*_(*t*) on the amount of S and/or D cells, as these factors may be produced by one and/or the other type of cells.

**Fig 5:**
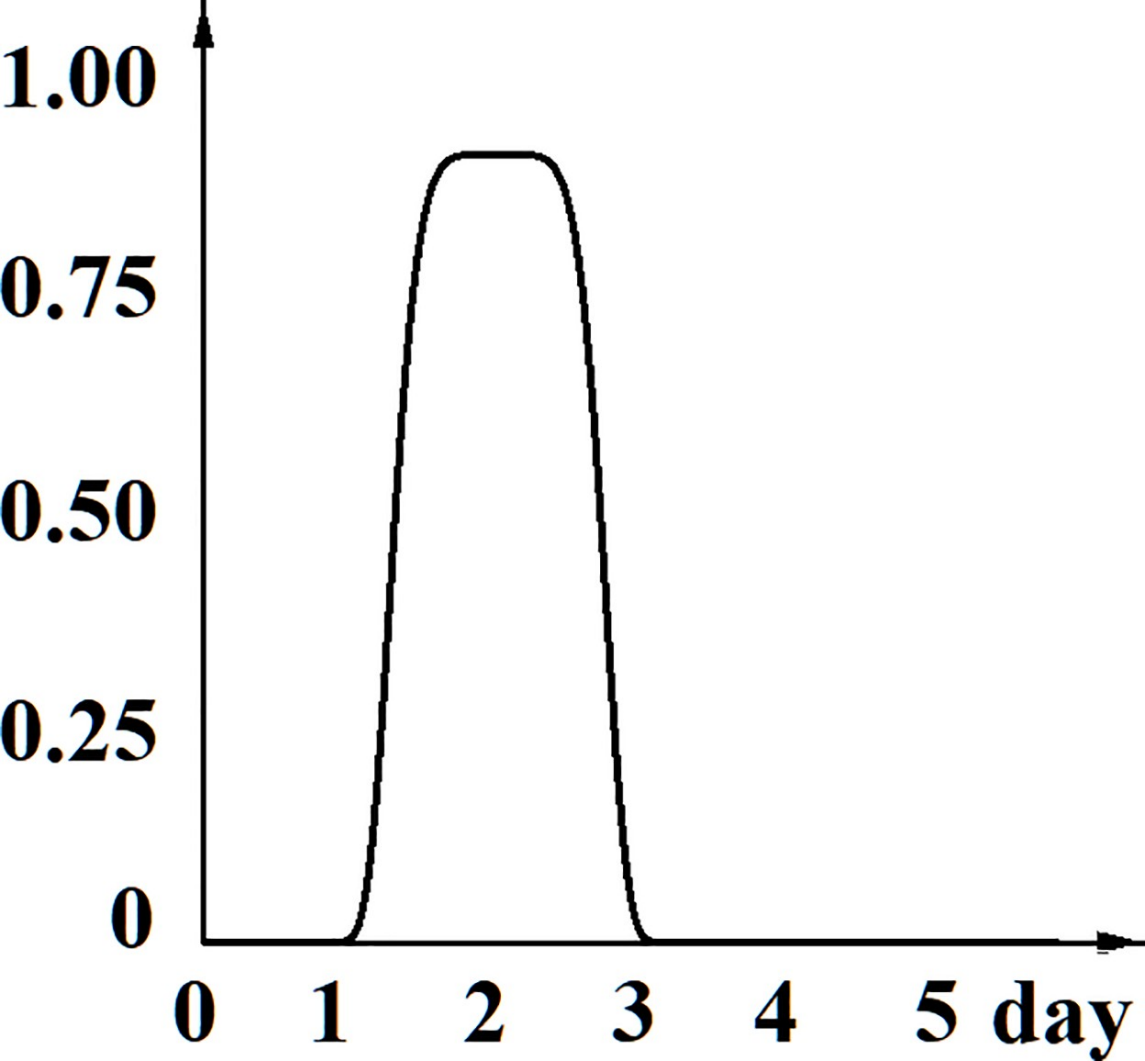
The function u(t) used to approximate the dynamics of underlying field factors.

Next, in order to determine the factors u_*k*_(*t*) which influence the evolution of probabilities p_i_(*t*) and q_i_(*t*), we will perform decomposition of given functions p_i_(*t*) and q_i_(*t*) over the functions u_*k*_(*t*). We will use the following form for the function u(t):

$$u(t)=be^{-a{\left(t-c\right)}^{m}},$$ (20)

where a is the coefficient reflecting the width of a particular curve u(*t*), b is its height, c is the position of the curve on the time axes, m is the shape (sharpness) of the curve (Fig 5).

This form, by varying the coefficients *a*,*b*,*c*,*m*, allows modeling various kinetics of factors, e.g., linear with different parameters, constant, exponential, trapezoid, etc.

According to formula (20), the dependence of probabilities *p*_*i*_,*q*_*i*_ on factors u_*k*_(*t*) can be written as:

$$y_{i}(t_{n})=\sum_{k=1}^{K}\left(b_{ik}e^{-a_{k}{\left(t_{n}-c_{k}\right)}^{m}}\right),$$ (21)

where *y*_*i*_, *i* = 1…6, is the generalized variable for all six probabilities *p*_1_,*p*_2_,*p*_3_,*p*_4_,*q*_1_,*q*_2_; K is the number of u_*k*_(*t*) factors considered, n = 1,…N is the time-points. Coefficients *a*_*k*_,*c*_*k*_ reflect details of a nature of a particular factor *k*, while coefficients *b*_*ik*_ reflect also the possibility of different influence of each factor *k* on the dynamics of each probability *y*_*i*_. Thus, by coefficients *b*_*ik*_ this variance of contribution of each factor u_*k*_(*t*) to each function of probability *y*_*i*_ is taken into account. We assume that the coefficient *b*_*k*_,

$$b_{k}=|{max}_{i}(b_{ik})|,$$ (22)

may correspond to relative concentration of a biochemical compound (factor u_*k*_) in a media.

Therefore we may define function u_*k*_(*t*) as

$$u_{k}(t)=b_{k}e^{-a_{k}{\left(t_{n}-c_{k}\right)}^{m}}$$ (23)

The test calculations and numerical simulation have shown that it is possible to set the value of the coefficient m = 6 for all factors, while coefficients *b*_*ik*_,*a*_*k*_,*c*_*k*_ should be selected by least-squares method. Thus, we will find such coefficients *b*_*ik*_,*a*_*k*_,*c*_*k*_ in the expression (21) that provides a minimum of the total quadratic deviation:

$$f=\sum_{n=1}^{N}\sum_{i=1}^{6}{\left(y_{i}(t_{n})-y_{in}\right)}^{2}\to min,$$ (24)

where *y*_*i*_(*t*_*n*_) are taken as in (21), while *y*_*in*_ correspond to a given set of unique functions of probabilities p_i_(t) and q_i_(t), determined from biological experiment and subsequent computations; *y*_*in*_ = p_i_(*t*_*n*_),*i* = 1…4, *y*_5*n*_ = q_1_(*t*_*n*_),*y*_6*n*_ = q_2_(*t*_*n*_).

For that it is necessary to ensure equality to zero of the expressions:

$$\left\{ \begin{array}{c} \frac{\partial f}{\partial a_{q}}=-2\sum_{n=1}^{N}\left(e^{-a_{q}{\left(t_{n}-c_{q}\right)}^{m}}{\left(t_{n}-c_{q}\right)}^{m}\sum_{i=1}^{6}b_{iq}(\sum_{k=1}^{K}\left(b_{ik}e^{-a_{k}{\left(t_{n}-c_{k}\right)}^{m}}\right)-y_{in})\right)=0\\ \frac{\partial f}{\partial c_{q}}=2a_{q}m\sum_{n=1}^{N}(e^{-a_{q}{\left(t_{n}-c_{q}\right)}^{m}}{\left(t_{n}-c_{q}\right)}^{\left(m-1\right)}\sum_{i=1}^{6}b_{iq}(\sum_{k=1}^{K}\left(b_{ik}e^{-a_{k}{\left(t_{n}-c_{k}\right)}^{m}}\right)-y_{in}))=0,\\ \frac{\partial f}{\partial b_{iq}}=2\sum_{n=1}^{N}(e^{-a_{q}{\left(t_{n}-c_{q}\right)}^{m}}(\sum_{k=1}^{K}\left(b_{ik}e^{-a_{k}{\left(t_{n}-c_{k}\right)}^{m}}\right)-y_{in}))=0 \end{array}\right.$$ (25)

where q = 1…K.

By this we obtain a system of Eq (25) for determining *b_ik_,a_k_,c_k_,k* = 1…*K*, *i* = 1…6, which was solved by the gradient descent method, starting with test initial values and obtaining the minimum of the function (24). The number of factors k is determined from the condition that the mean deviation *y_i_*(*t_n_*) from *y_in_* should not exceed a given value of permissible variation *ε_p_*:

$$\varepsilon \equiv \frac{\sum_{n=1}^{N}\sum_{i=1}^{6}\left|y_{i}(t_{n})-y_{in}\right|}{6N}100\%<\varepsilon_{p}.$$ (26)

The search starts from suggested initial number of factors K, and in the case when condition (26) is not fulfilled for a given *ε*_*p*_, should be repeated for K+1, and further on until the satisfaction of (26).

**Fig 6:**
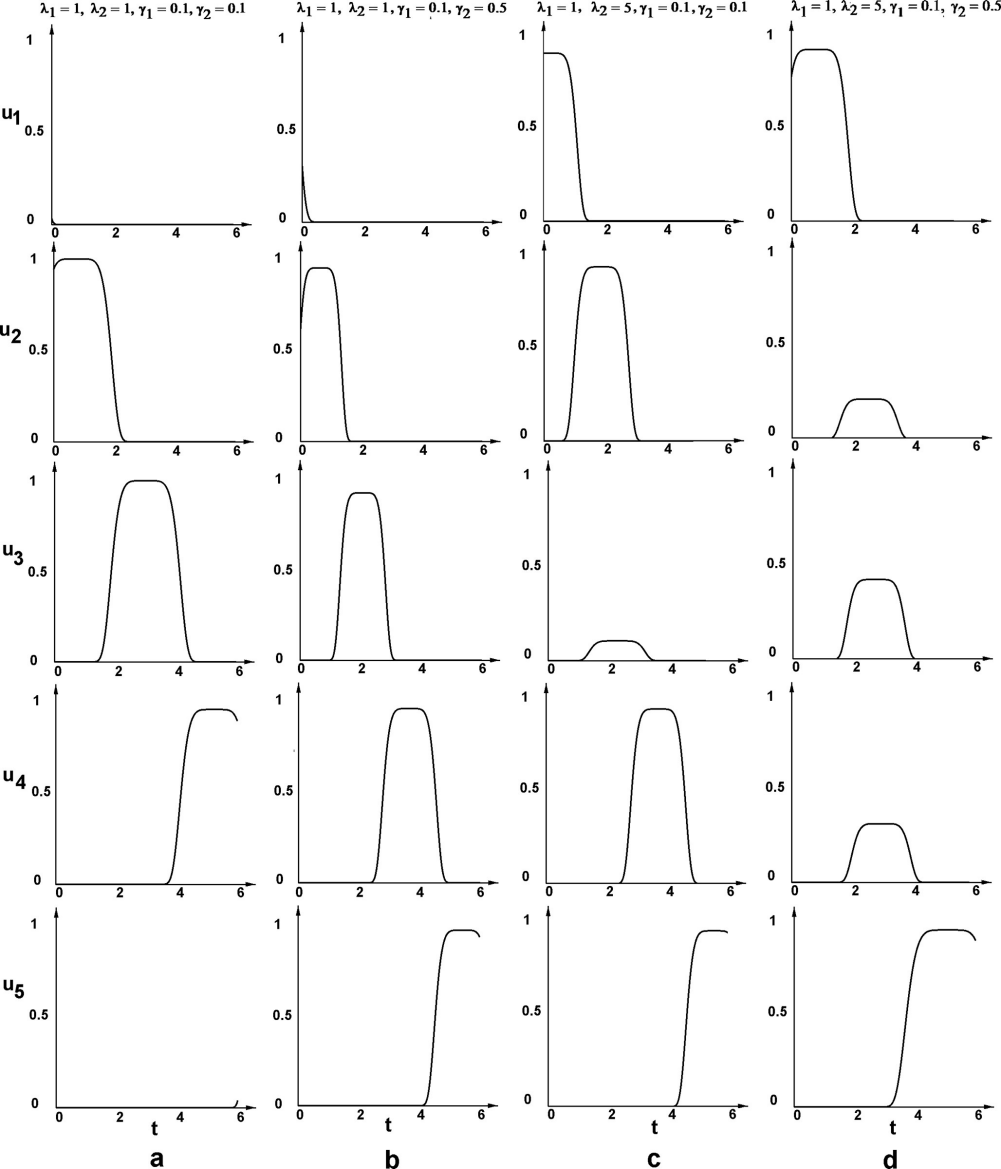
Underlying field u_*k*_(*t*) functions for four considered sets of λ_i_,γ_i_. The height of each factor u_*k*_(*t*) corresponds to *b*_*k*_.

Next a corresponding computer program, automatically performing all necessary computations for a chosen *ε*_*p*_, was developed and applied for the particular set of curves *p*_1_(*t*),*p*_2_(*t*),*p*_3_(*t*),*p*_4_(*t*),*q*_1_(*t*),*q*_2_(*t*) presented on the Fig 3 (the corresponding algorithm and the program are provided in Methods section). The results of computations, determining the number and pattern of u_*k*_(*t*) factors, providing the satisfaction of the condition ε ≤ 1% for each experimental case (the same sets of λ_i_,γ_i_ considered before), are presented on Fig 6, where the height of each factor *b*_*k*_.

The search started from suggested initial number of factors *K* = 4, and has elucidated the most plausible number of major u_*k*_(*t*) factors for each experimental case (which varied from 3 to 6 factors depending on the case), together with their specific patterns (shape, height and timing).

It was found that in 3 cases out of 6 explored, the starting number of factors *K* = 4 was enough to determine a set of essential influencing field factors u_*k*_(*t*) with sufficiently good value of variation ε.

Moreover, in two cases, namely, for the set λ_1_ = 1,λ_2_ = 1,γ_1_ = 0,1,γ_2_ = 0,1 (ε = 0,49%; Fig 6A) and for the set λ_1_ = 1,λ_2_ = 10,γ_1_ = 0,1,γ_2_ = 0,1 (ε = 1%; not shown), the program has detected only 3 factors as essential, thus showing the capability to find the minimal possible set of factors u_*k*_(*t*) for the best fitting, independent of the starting number of factors K. In one case 4 factors were determined: λ_1_ = 1,λ_2_ = 1,γ_1_ = 0,1,γ_2_ = 0,5 (ε = 0,47%, Fig 6B). For the other 3 cases, for which with *K* = 4 unsatisfactory ε value was obtained, the search was continued with greater number of factors *K* up to optimal of fitting. Finally, for two cases the final number of essential influencing factors was 5: λ_1_ = 1,λ_2_ = 5,γ_1_ = 0,1,γ_2_ = 0,1 (ε = 0, 82%; Fig 6C) and λ_1_ = 1,λ_2_ = 5,γ_1_ = 0,1,γ_2_ = 0,5 (ε = 1,0%; Fig 6D), while in one case, for which K = 5 gave ε = 1,2, the final number of factors K appeared to be equal to 6: λ_1_ = 1,λ_2_ = 10,γ_1_ = 0,1,γ_2_ = 0,5 (ε = 0, 75%; not shown).

**Fig 7:**
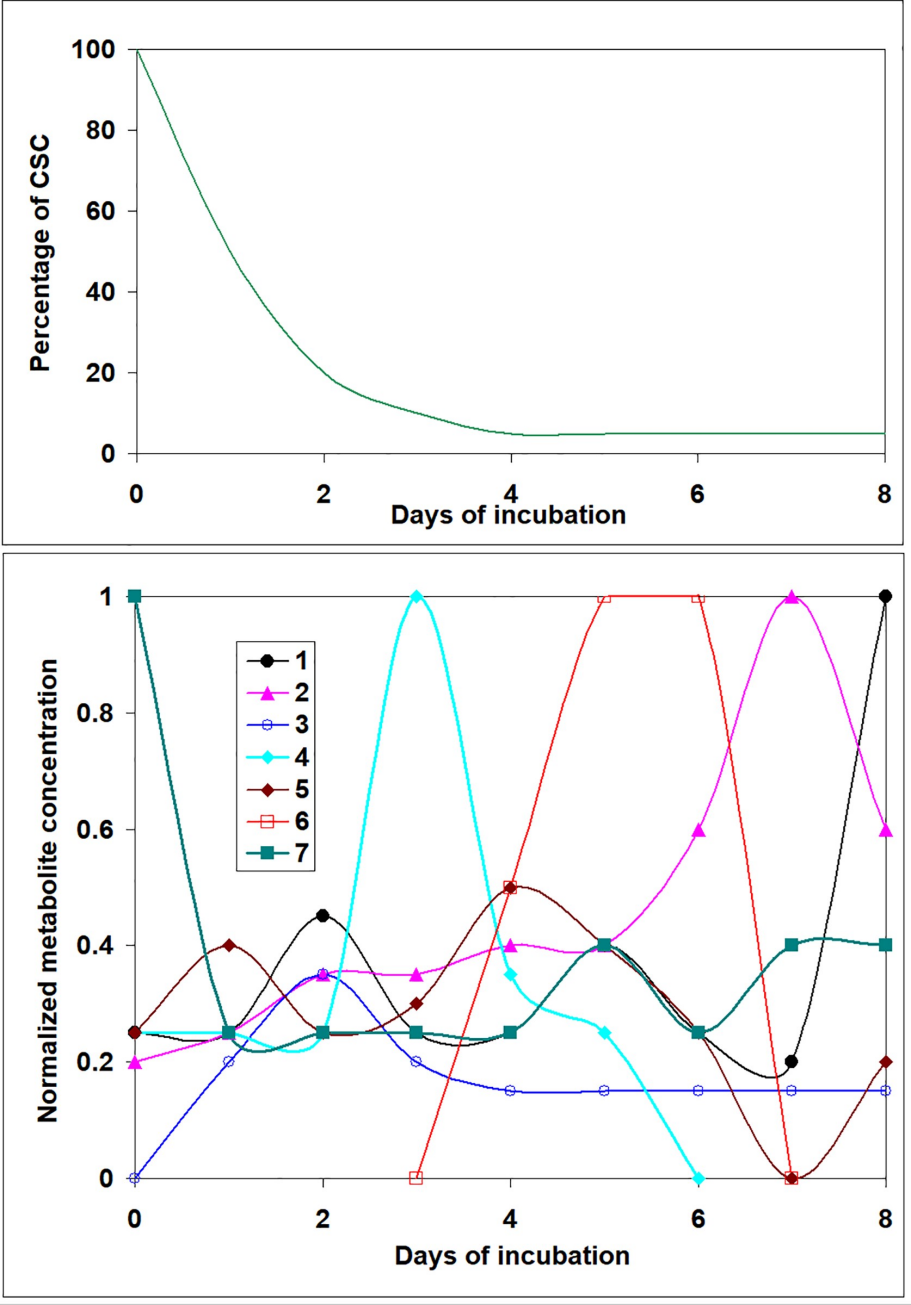
An example of suggested Metabolome (Secretome) time–dependent experimental data required to detect a set of underlying field factors by using the proposed mathematical modeling. In the presented illustration, metabolites *N4* and *N6* have kinetics in agreement with the ones found for factors u_3_(*t*) and u_4_(*t*) in Fig 6A.

We expect that this model can give insight into a search for biochemical factors involved in controlling cell behavior. For example, if in a course of measurement of CSC population kinetics, also the Metabolome (Secretome) profiles of the culture media can be obtained, the secreted factors which appear, increase, decrease or disappear at each time point can be identified (Fig 7).

Next predictions on u_*k*_(*t*) factors obtained by mathematical model for a given case (*s*(*t*), λ_i_, γ_i_) (Fig 6) should be compared with the results of the corresponding Secretome profile (Fig 7). The biochemical factors having the kinetics and patterns coinciding with the predicted underlying field functions u_*k*_(*t*) could be the good candidates for being involved in controlling cell behavior.

It is probable that out of possibly several hundreds metabolites in Secretome, several will fit to predicted u_*k*_(*t*). In this case two variants may be envisioned: either a cumulative effect of all (or several) indicated metabolites creates the u_*k*_(*t*) signal, or only one among them has this special effect.

*Important Remark*. Though the system of Eq (25) cannot be solved uniquely, our computational experiments have shown that by requesting *ε* to be considerably small and by setting the approximation time steps in the program to be 10^−4^ arbitrary units, we get minimizing of the area of all u_*k*_(*t*) in such a way that the difference between the various solutions is negligible, as it is below the sensitivity of experimental equipment used for monitoring the factors. For example, for *ε*≈1%, the maximal variation of coefficient b reflecting a concentration of a factor in a media, is 3%, and maximal variations of coefficients a and c reflecting the time of existing of the factor in media are 3%.

This means that the model suggests an approach to determine the secreted factors influencing the time-dependent cells behaviors leading to the stabilization of CSC population.

### Predictions of putative production of secreted factors u_k_(t) by S or D cells

The identification of the cell type, stem or non-stem, producing the factor(s) influencing CSC behavior might be important for basic research as well as for potential medical applications. In order to provide a tool for such a prediction, we assumed that some factors u_k_(t) can be produced (completely or mostly) by S cells, others by D cells, while some factors may be without explicit belonging to any type of these cells.

To account for this possibility, we have considered a set of functions U_kc_(t) which provides discriminatory dependence of a factor u_k_(t) on S or D cells kinetics:

$$U_{kc}(t)=u_{k}(t)\;M_{c},\;k=1\ldots K,\;c=1\ldots 3,$$ (27)

where *M*_*c*_ is a set of three functions: *M*_*c*_ = {s(t),d(t),1}.

This means that having an optimal set of factors u_*k*_(*t*), found at the previous step (Fig 6) for a given set of parameters λ_i_,γ_i_ and given kinetics p_i_(*t*), q_i_(*t*),we will consider all variants of multiplication of each function u_*k*_(*t*) on each of the functions s(t),d(t), and on the constant equal to 1, with determining *ε* value for each U_kc_(t) according to formula (26).

We can assume that the result of such a multiplication that gives the smallest *ε* value represents the possible dependence of the factors upon the types of cells (*S* or *D* cells). The variant of multiplication on 1 means independence of a factor on *S* or *D* kinetic, which can reflect the production of these factors evenly by both types of cells or their production by the environment.

The results of the computation U_kc_(t) for two selected cases are presented in the Table 2.

**Table 2:** Possible dependence of the factors u_k_ upon the type of producing cells (S or D cells).

(A) λ_1_ = 1,λ_2_ = 5,γ_1_ = 0,1,γ_2_ = 0,1					(B) λ_1_ = 1,λ_2_ = 10,γ_1_ = 0,1,γ_2_ = 0,5				
u_1_	u_2_	u_3_	u_4_	*ε*(%)	u_1_	u_2_	u_3_	u_4_	*ε*(%)
S	D	D	S	1.298	S	D	1	S	1.013
**1**	**1**	**1**	**1**	**1.373**	S	D	D	S	1.015
S	D	S	D	2.013	S	1	S	D	1.236
S	S	D	D	2.036	S	1	D	S	1.236
D	D	S	S	2.644	S	1	1	S	1.274
D	D	D	S	2.741	S	1	S	1	1.275
D	D	D	D	4.249	S	D	S	S	1.321
S	S	S	S	4.91	1	1	S	S	1.33
D	1	1	1	7.379	1	D	S	S	1.338
					1	S	1	1	1.361
					1	S	1	D	1.362
					1	1	S	1	1.366
					1	1	S	D	1.368
					1	S	D	1	1.372
					1	S	D	D	1.373
					S	S	D	D	1.373
					S	S	D	1	1.376
					1	1	1	S	1.38
					1	1	D	S	1.385
					1	D	S	1	1.388
					1	D	S	D	1.39
					1	D	1	S	1.398
					S	D	S	1	1.398
					S	D	S	D	1.4
					1	D	D	S	1.401
					**1**	**1**	**1**	**1**	**1.435**
					S	S	S	S	5.211
					D	1	D	1	6.516

Two chosen experimental cases (A) and (B) for different sets of parameters λ_1_,λ_2_,γ_1_,γ_2_ are presented, with four secreted factors u_*k*_ for each of them. In Table 2, “S” or “D” means considering the dependence of a factor on *S* or *D* cells kinetics, while “1” means considering an independence of a factor on any specific type of cells.

The variant (1,1,1,1) marked in bold corresponds to the independence of all factors upon any specific type of cells. All variants with better results (*ε* is smaller than in (1,1,1,1)) and some chosen variants for the worse results (*ε* is larger than in (1,1,1,1)) are shown.

It can be seen that for the case (A): λ_1_ = 1,λ_2_ = 5,γ_1_ = 0,1,γ_2_ = 0,1, for K = 4 and without differentiating multiplication (corresponding to the variant 1,1,1,1) *ε* = 1.373% (Table 2A). The computation has shown that all other variants of multiplication give worse results (some selected ones are shown), except for only one variant (*S*,*D*,*D*,*S*), though decreasing the value of *ε* not considerably (*ε* = 1.298%). However, it still can be an indication of differential production of the factors (*t*) by *S* or *D* cells.

Much stronger dependence on *S* and *D* kinetics is demonstrated in the other case (B): (A) λ_1_ = 1,λ_2_ = 10,γ_1_ = 0,1,γ_2_ = 0,5, where for *K* = 4 and without differentiating multiplication the lowest *ε* value was found as *ε* = 1.435%, while in the best variant with differentiating multiplication (*S*,*D*,1,*S*), it decreases up to 1.013 (Table 2B). Also, the fact that in this case 25 variants with differentiating multiplication give better results than the variant without considering the dependence on *S* or *D* kinetics (1,1,1,1), can indirectly point on the necessity of concluding this additional calculation in this concrete case. On the other hand, this fact can be an indicator of instability of the found best variant of multiplication (for this case, all variants with better results (*ε* is smaller than in (1,1,1,1)) and some chosen variants for the worse results (*ε* is larger than in (1,1,1,1)) are shown in Table 2B).

It is important to note, that in order to show a larger difference in multiplicative and non-multiplicative outcome, we present in Table 2 the results of a differentiating multiplication, which was started from the optimal sets of non-multiplied factors u_*k*_(*t*) obtained with the requirement *ε*≤1.5% corresponding to *K* = 4. The program started from the best non-multiplicative set obtained in stricter requirement *ε*≤1% (presented on Fig 6, and corresponding to K = 5 factors in the case (A) and K = 6 in the case (B)), gives the same qualitative results, i.e., the same dependence of particular factors on *S* and *D* kinetics, but with less significant decrease of *ε* value for the resultant best multiplication.

This means that in order to obtain reliable results about the dependence of the underlying field factors on *S* or *D* cells, one should apply the suggested computational program starting with the optimal sets of non-multiplied factors u_*k*_(*t*) obtained for possibly high level of *ε*_*p*_.

## Conclusions

Using a set of theoretical assumptions and basic knowledge of cancer cells population behavior, we suggest a mathematical approach which may help experimentalists gain insight into a broad array of cancer cell fates using a limited amount of experimental measurements. The computational program based on our model allows determining several important characteristics of CSC behavior in a cancer cell population.

First, on the basis of a minimal set of experimentally measured values (rates of cell division, of cell death and CSC population kinetics s(*t*)), our model enables the prediction of probabilities of cell-line specific modes of *S* and *D* cells divisions, including the unusual *D*→*S* transition. Second, the model explores the dynamics of cell-cell interaction factors u_k_(t) influencing cancer cells behavior (namely, the time-dependent probability of cell division modes p_i_(t) and q_i_ (t)). Finally, the model allows to concern the likelihood of production of these factors u_k_(t) by S (stem) or D (non-stem) cells in the cancer cell population.

A potential drawbacks of the proposed model is that the key assumptions of the model are pure theoretical and that the model is rather simple. For example, we do not take into the account the effects of deterministic-stochastic interplay which was first considered for CSC modeling in [37] and next developed in [58]. Another obvious limitation of the model is that the suggested applications of it still have to be confirmed by biological experiments.

However, we can anticipate the development of several epistemological and practical applications regarding cancer cell behavior, which cannot be accomplished by using biological methods only. As an example, the proposed work may give insight into evaluation of the risks of cancer to relapse upon radio- or chemotherapy, based on the experimental measurement of its CSC kinetics. Also, it may help to find the treatment achieving the most efficient suppression of cancer subpopulations, as well as the schedule achieving the best therapeutic results by considering the predicted *underlying field* behavior. An essential part of this schedule may depend on identifying the treatment time points at which elimination of specific underlying field factors will be predicted as crucial for cancer population abolishment.
